# Health Care Quality Improvement for ST-Segment Elevation Myocardial Infarction: A Retrospective Study Based on Propensity-Score Matching Analysis

**DOI:** 10.3390/ijerph18116045

**Published:** 2021-06-04

**Authors:** Junxiong Ma, Xuejie Dong, Yinzi Jin, Zhi-Jie Zheng

**Affiliations:** 1 Department of Global Health, School of Public Health, Peking University, Beijing 100191, China; 1310306228@bjmu.edu.cn (J.M.); dongxj@bjmu.edu.cn (X.D.); zhengzj@bjmu.edu.cn (Z.-J.Z.); 2 Institute for Global Health, Peking University, Beijing 100191, China

**Keywords:** chest pain center, quality improvement, STEMI

## Abstract

Reducing the treatment delay by organizing delivery of care on a regional basis is a priority for improving the quality of ST-segment elevated myocardial infarction (STEMI) care. This study aimed to evaluate the impact of the combined measures on quality metrics of healthcare delivery in Suzhou. The data were collected from the National Chest Pain Center (CPC) Data Reporting Database. 4775 patients were recruited, and after propensity-score matching, 1078 pairs were finally included for analysis. We examined the changes in quality metrics of care including prehospital and in-hospital processes, and clinic outcomes. Quality improvement (QI) implementation improved most process indicators. However, these improvements did not yield decreased in-hospital mortality. The door-to-balloon and the FMC-to-device time decreased from 85.0 and 98.0 min to 78 and 88 min, respectively (*p* < 0.001). Cases transferred directly via EMS had a greater improvement in most of process indicators. The proportion of patients transferred directly via EMS was 10.3%, much lower than that of self-transported patients at 58.3%. Tertiary hospitals showed greater performance improvement in process indicators than secondary hospitals. The percentage of cases using EMS remained low for suburban areas. The establishment of coordinated STEMI care needs to be accompanied with solving the fragmented situation of the prehospital and hospital care, and patient delay should be addressed, especially in suburban areas and on transferred-in inpatients.

## 1. Background

ST-segment-elevation myocardial infarction (STEMI) is the deadliest acute cardiac event and requires rapid coordination of care beginning at the time of symptoms’ onset, and percutaneous coronary intervention (PCI) within 120 min of onset is the typically recommended treatment [1]. Despite the widespread promulgation and endorsement of the guidelines, their translation into clinical practice remains suboptimal. The time from onset to PCI is approximately 290 min, and only 7% of patients receive timely PCI therapy, contributing to increased mortality from cardiovascular disease, which is the leading cause of death in China [2]. The times from call-to-emergency medical services (EMS) to the scene and the door-to-balloon time are about 21 and 94 min respectively, which is longer than the average time of 7 and 59 min in some developed countries [3,4]. Clinical studies have shown that for every 30 min that treatment is delayed, the one-year mortality rate after STEMI increases by 7.5% [5,6]. Therefore, reducing the treatment delay by organizing delivery of care on a regional basis is a priority for improving the quality of STEMI care in China.

Hospital-based clinical registries and the related quality improvement (QI) initiatives can facilitate the delivery of effective healthcare [7,8]. This belief is supported by several prior efforts in many developed countries [9,10,11]. The development of a chest pain center (CPC) is a foundation for establishing delivery systems of healthcare for acute cardiac events. However, there has been no national unified registry for garnering hospital participation, standardizing clinical practices, and facilitating QI initiatives in China [12]. To address this need, with the support of the National Health and Family Planning Commission of China (NHFPC, renamed to the National Health Commission or NHC in 2018), the Chinese Society of Cardiology (CSC) officially issued the China CPC Accreditation Criteria in 2013. Afterwards, the NHFPC enacted the “Notice on Strengthening the Capacity of Healthcare Delivery for Acute Cardiac Events” in 2015. The official notice called for establishing regional collaborative healthcare networks through integrating community-, prehospital- and hospital-care for managing acute cardiac events. Under these directives, hospital-based CPCs were quickly developed throughout China, forming regional networks of multidisciplinary specialized cardiac care centers.

Suzhou was the first city to respond to the national call, and has implemented the multifaceted QI initiatives that can facilitate the delivery of effective healthcare since 2016. The QI initiatives include: (i) accreditation of hospital-based CPCs, (ii) establishing a unified hospital-based clinical registry for quality monitoring and assessment, (iii) providing ongoing quality reviews and feedback, and (ⅳ) organizing education and training activities aimed at healthcare professionals. Suzhou was the first city to establish the Medical Priority Dispatch System (MPDS) to guide standardized prehospital EMS at a regional level.

Moreover, the EMS in Suzhou has taken the lead in establishing an information sharing system by linking the MPDS and the hospital-based CPC registry, to integrate the prehospital and hospital care, and to facilitate the coordination of care at the time of entering the EMS system.

The EMS system in China includes prehospital emergency centers that provide prehospital care, and hospital emergency departments and the intensive care units that provide hospital care [13]. In most parts of China, the prehospital and in-hospital processes are separately managed by emergency centers and hospitals [14]. Hence, connecting prehospital and in-hospital processes is crucial for STEMI treatment, and includes patterns of transport to the hospital, the transfer from secondary hospitals (with basic CPCs) to tertiary hospitals (with combined CPCs), and transfer from suburban to urban hospitals. The EMS in Suzhou is unique in that it has focused on the establishment of regional systems of STEMI care by facilitating the coordination between prehospital emergency centers and hospital emergency departments, and the hierarchical delivery between secondary and tertiary hospitals.

Therefore, it is warranted to evaluate the implementation of the combined measures in Suzhou, including the combined QI initiatives and the establishment of the information sharing system between the MPDS and the hospital-based CPC registry. Building on efforts in establishing a unified hospital-based clinical registry, we developed the first prospective study in China: (1) to evaluate the impact of the combined measures focusing on establishment of regional systems of STEMI care on quality metrics of healthcare delivery in terms of the care processes and clinical outcomes; and (2) to compare the impacts between secondary and tertiary hospitals, among patients with different modes of transfer, and between urban and suburban areas.

## 2. Methods

### 2.1. Study Site

The study was conducted at all of the 40 accredited hospital-based CPCs in the nine districts of Suzhou. Suzhou is located in the east of China, has a population of 10.72 million, ranking 13th in China in terms of population size, with a gross domestic product per capita of ¥173,456.4 yuan ($25,161.8 USD), ranking 5th among the total 661 cities in mainland China in 2019. The operation of the QI initiatives was carried out and managed by the Management Board established by the CSC under the authorization of the NHC nationwide. The Data Management Committee, one of the committees of the Management Board was responsible for evaluating and monitoring the QI initiative. All the accredited CPCs in Suzhou were instructed to submit consecutive eligible patients to the China CPC Data Reporting Database (http://data.chinacpc.org/, accessed on 6 September 2019), a national surveillance system for monitoring the characteristics, treatments, and outcomes of patients diagnosed with STEMI. Each hospital is responsible for its own data collection.

### 2.2. Data Collection

Data on all patients older than 18 years with a final diagnosis of STEMI at discharge in Suzhou were drawn from the China CPC Data Reporting Database. 4775 patients were recruited consecutively in the 40 hospital-based CPCs from 1 April 2016 to 31 March 2019. The combined measures including QI initiative implementation and accreditation were applied at the hospital level after hospital accreditation. So the pre-combined measures were cases included in the hospital which had not been accredited before the combined measures, and the post-combined measures were cases included in the hospital which had been accredited after the combined measures. 513 patients were excluded if they died before or within 10 min of hospital arrival [15,16], or if a contraindication was documented as the reason for withholding the program; thus, 4262 patients were included in the study. After propensity-score matching (PSM) by controlling the confounding factors (Supplementary Table S1) [17], 1078 pairs were finally included for analysis pre- and post-combined measures.

### 2.3. Measure Definitions

Quality metrics included prehospital processes, in-hospital processes, and outcome indicators (Supplementary Table S2), which were used as the core set of quality indicators for measuring CPC performance in the quarterly and annually benchmarked reports, developed by the China CPC Headquarters. The quality metrics were key performance indicators, based on the ACC/AHA Performance Measures and Class I Recommendations from the most updated ACC/AHA clinical practice guidelines. Accredited CPCs should continuously report data for monitoring and feedback to the China CPC Data Reporting Database. Improvements in adherence to the guideline recommendations are facilitated through monthly and quarterly hospital-specific performance feedback reports.

### 2.4. Statistical Analysis

We assessed the changes in the quality metrics of STEMI care pre- and post-combined measures. We also compared the changes in quality metrics between secondary and tertiary hospitals, among patients who had different modes of transfer, and between suburban and urban areas. Changes in quality metrics were assessed using univariate analyses, including the Kruskal–Wallis test, chi-square test, *t*-test and one-way analysis of variance. Fisher’s exact test was used to compute 95% confidence intervals for each quality metric. *p*-values < 0.05 were considered statistically significant. All statistical analyses were conducted in R software (R Foundation for Statistical Computing, Vienna, Austria, and Version 3.6.3).

### 2.5. Patient and Public Involvement

Patients or the public were not involved in the design, or conduct, or reporting, or dissemination plans of our research.

## 3. Results

### 3.1. Characteristics of STEMI Patients before and after the PSM

Before the PSM, patients enrolled after the combined measures (post-combined measures patients) were relatively younger (60.40 ± 14.65 years vs. 61.91 ± 14.97 years, *p* = 0.004) than those enrolled before the combined measures (pre-combined measures patients). Post-combined measures patients were less likely to have sustained chest pain (77.2% vs. 77.3%, *p* = 0.001) or intermittent chest pain (22.2% vs. 14.6%, *p* < 0.001), and relief of chest pain had a higher percentage (4.4% vs. 2.5%, *p* = 0.001). Regarding vital signs, the heart rate per minute was slightly lower (78.56 ± 18.23 vs. 77.07 ± 19.61, *p* = 0.021) in the post-combined measures patients. After the PSM, the mirrored histograms before and after matching are shown in Figure S1 to present the propensity score distribution. the standard deviation of all covariates was <2%, indicating no significant differences between the two groups in demographics, chest pain symptoms, vital signs or Killip grading (Table 1).

### 3.2. Quality Metrics before and after Combined Measures

**Figure 1:**
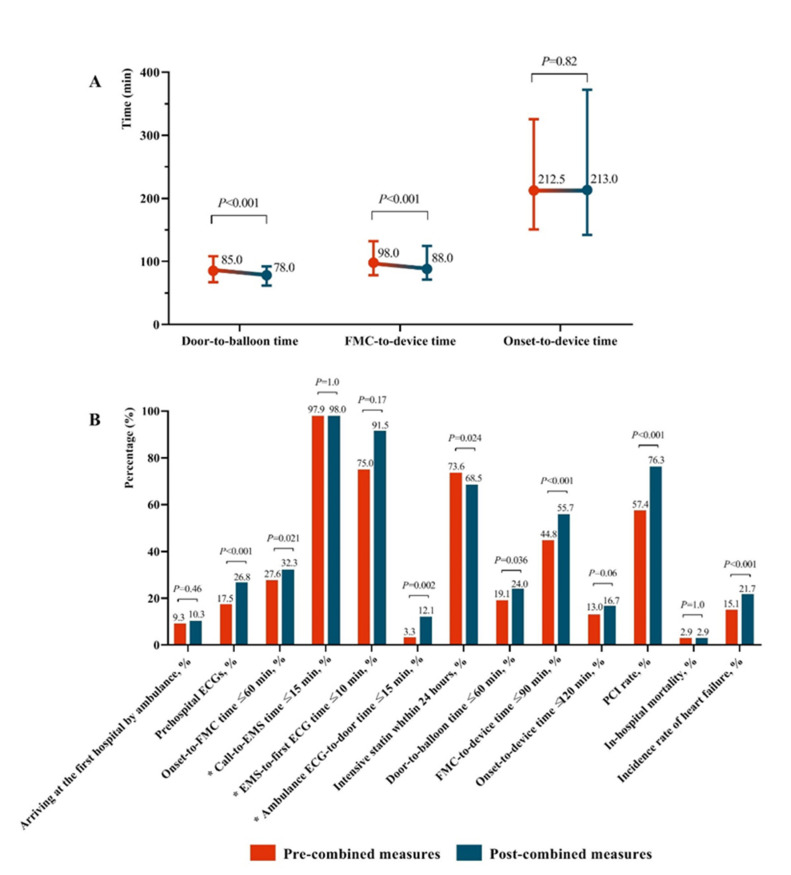
Comparison of pre- and post-combined measures after propensity-score matching (* For ambulance transported cases). (**A**): Time change of pre- and post-combined measures; (**B**): Rate change of pre- and post-combined measures.

Figure 1 shows the changes in reperfusion time and in-hospital mortality before and after the combined measures. The median total time (q1, q3) from symptom onset to PCI did not significantly change from 212.5 (150.8, 325.5) to 213.0 (142.0, 372.0) minutes. However, the door-to-balloon and the median FMC-to-device (q1, q3) time decreased from 85.0 (67.0, 108.0) and 98.0 (78.0, 132.8) minutes to 78 (61.5, 92.0) and 88 (71.0, 124.5) minutes, respectively (*p* < 0.001). The percentage of patients meeting guideline goals increased significantly, except for the percentage of patients arriving at the first hospital by ambulance (14.6% vs. 12.2%, *p* < 0.001), the proportion of call-to-EMS time ≤15 min for ambulance-transported cases (45.6% vs. 47.8%, *p* = 0.622) and the proportion of EMS-to-first electrocardiogram (ECG) time ≤ 10 min for ambulance-transported cases (75.0% vs. 91.5%, *p* = 0.171). The in-hospital mortality persisted before and after the combined measures (2.9% vs. 2.9%, *p* = 1.000).

### 3.3. Comparisons between Tertiary and Secondary Hospitals

We compared the changes in reperfusion time and in-hospital mortality before and after the combined measures by tertiary and secondary hospitals (Table 2). For prehospital processes, although most indicators of the secondary hospitals did not show improvement after the combined measures, the tertiary hospitals exhibited significant increases in some indicators, including the rate of prehospital ECGs (21.4% vs. 27.1%, *p* = 0.006), the proportion of onset-to-FMC (EMS arrival or walk-in to emergency department) time ≤ 60 min (27.8% vs. 32.3%, *p* = 0.047) and the proportion of ambulance ECG-to-door time ≤ 15 min for ambulance-transported cases (2.4% vs. 12.2%, *p* = 0.001).

For in-hospital processes, most indicators of tertiary hospitals improved significantly after the combined measures, including the proportion of door-to-balloon time ≤ 60 min (18.9% vs. 24.2%, *p* = 0.029), the proportion of FMC-to-device time ≤ 90 min (44.8% vs. 55.8%, *p* < 0.001), the door-to-balloon time (85.0 [66.8, 108.3] min vs. 78.0 [61.3, 92.0] min, *p* < 0.001), the FMC-to-device time (98.0 [78.0, 132.3] min vs. 88.0 [71.0, 124.8] min, *p* < 0.001), and the PCI rate (70.8% vs. 76.9%, *p* = 0.003).

Regarding outcome indicators, the in-hospital mortality decreased in both the tertiary and secondary hospitals, although not significantly. However, the heart failure incidence rate increased significantly in the tertiary hospitals (13.4% vs. 21.9%, *p* < 0.001).

### 3.4. Comparisons between Different Patterns of Transfer

We compared the changes in quality metrics before and after the combined measures among walk-in patients, in-hospital patients, those transferred directly via EMS, and those transferred from other hospitals (Table 3). For walk-in patients, most indicators improved significantly after the combined measures, including the proportion of onset-to-FMC time ≤ 60 min (22.6% vs. 29.7%, *p* = 0.004), the proportion of door-to-balloon time ≤ 60 min (8.8% vs. 17.2%, *p* = 0.001), the proportion of FMC-to-device time ≤ 90 min (55.0% vs. 69.8%, *p* < 0.001), and the proportion of onset-to-device time ≤ 120 min (12.3% vs. 19.8%, *p* = 0.005), as well as the door-to-balloon time (89.0 [75.0, 120.0] min vs. 82.0 [68.0, 99.3] min, *p* < 0.001), the FMC-to-device time (88.00 [73.0, 119.0] min vs. 80.00 [66.0, 98.0] min, *p* < 0.001), and the PCI rate (52.7% vs. 78.2%, *p* < 0.001).

For patients transferred to the hospital directly via EMS, most indicators improved significantly, including the proportion of onset-to-FMC time ≤ 60 min (39.6% vs. 62.9%, *p* = 0.002), the door-to-balloon time (87.0 [72.0, 109.0] min vs. 71.0 [60.0, 88.0] min, *p* = 0.005), the FMC-to-device time (102.5 [82.3, 128.3] min vs. 85.0 [68.0, 105.0] min, *p* = 0.004), and the onset-to-device time (175.0 [140.0, 254.0] min vs. 141.5 [115.3, 175.8] min, *p* = 0.010).

For transferred-in patients, the onset-to-device time (240.0 [171.0, 347.0] min vs. 277.00 [193.0, 465.0] min, *p* = 0.004) increased significantly after the combined measures. The door-to-balloon time (65.0 [47.8, 84.5] min vs. 71.5 [49.0, 87.0] min, *p* = 0.158) and the FMC-to-device time (121.5 [98.3, 161.8] min vs. 136.0 [99.0, 185.0] min, *p* = 0.055) also increased, but not significantly.

### 3.5. Comparisons between Urban and Suburban Areas

We compared the changes before and after combined measures between urban and suburban areas (Table 4). Pre-combined measures data showed that all in-hospital indicators were better in urban hospitals than in suburban hospitals, except the rate of intensive statin use within 24 h. In both urban and suburban hospitals, most indicators improved significantly after the combined measures, including the rate of prehospital ECGs, the proportion of onset-to-FMC time ≤ 60 min (EMS arrival or walk-in to emergency department), the proportion of FMC-to-device time ≤ 90 min, the door-to-balloon time, the FMC-to-device time, and the PCI rate. However, for urban areas, the rate of intensive statin use within 24 h (67.7% vs. 54.3%, *p* < 0.001) decreased, and the heart failure incidence rate (14.2% vs. 24.5%, *p* < 0.001) increased. For suburban areas, the percentage of patients arriving at the first hospital by ambulance (10.7% vs. 5.5%, *p* < 0.001) decreased significantly.

## 4. Discussion

Many studies have shown that the transmission of prehospital response is an important base for bypassing the emergency department and CCU. It is also an important way to reduce the in-hospital delay and is of great significance in shortening the treatment time, which is consistent with the Suzhou results [7,18]. The EMS of Suzhou represents the combined measures for translating efficacy into effectiveness as narrowing the evidence-based gap in the treatment of STEMI. The EMS of Suzhou focuses on the establishment of regional systems of STEMI care that can be adapted to the context of China’s health system. This study, to our knowledge, is the first retrospective study to examine the impacts of the regionalization of STEMI care, including the combined QI initiatives and the establishment of the information sharing system between the MPDS and the hospital-based CPC registry. The findings have implications for further promoting the measures, with the ongoing engagement of QI efforts and for their potential implementation in low- and middle-income settings where the burden of STEMI is increasing at an unprecedented rate [19].

We found that implementing the combined measures improved many process indicators significantly. However, these improvements did not translate into better clinical outcomes. The results are consistent with findings from the recent systematic review that analyzed 32 studies (randomized controlled trials and nonrandomized quasi-experimental studies) from 5858 records for QI interventions on process of care measures and clinical outcomes [20]. Although there is substantial heterogeneity in the QI interventions, a range of studies show that certain interventions are associated with better processes of delivering STEMI care [21,22,23]. In our study, the most important finding is that there was a significant improvement in the in-hospital service quality. We found that although the in-hospital mortality rate and onset-to-device time did not change significantly, the time from FMC to device was significantly shortened. The achievements can be attributed to coordination of care at a regional level, which has gained support and been incorporated into national guidelines for CPC accreditation.

Generally, it is important for us to coordinate emergency services systems and population campaigns to raise awareness about STEMI management and avoid preventable delay. The key elements of our efforts included broad regional leadership, highly-developed eHealth technology [24], and well-organized coordinators. First, a nationwide collaborative network which led the implementation and supervision of the QI initiatives is well organized by the CSC under the authorization of the NHC. The operational structure consists of the Management Board and the Executive Board, between which the the China CPC Headquarters is the link, located in Suzhou. The China CPC Headquarters is committed to monitoring the implementation of the QI initiatives through continuous audit and feedback on the China CPC Data Reporting Platform. The China CPC Headquarters creates practical tools for sites to help improve the quality of data reporting and consistency across the registered hospitals. Examples of these tools include guidelines for data reporting, a set of internal quality assurance tools, and a yearly data audit program. Secondly, with the support of the NHC of Suzhou, the online software, mobile APPs and other eHealth technology were developed for real-time data reporting, to improve data quality and reporting efficiency. Suzhou took the lead in establishing the MPDS and the information sharing system by linking the MPDS and the hospital-based CPC registry, to facilitate the coordination of care at the time of entering the EMS system. STEMI patients can be transferred to an appropriate hospital based on disease conditions with the support of the MPDS, and can be guided through first aid by the first bystander before the ambulance arrives by the tele-guide of the dispatcher. The prehospital ECGs on the ambulance can be transmit to the hospital through the online software, thereby allowing transfer to the Cath Lab, bypassing the emergency department and coronary care unit. Third, there are well-organized coordinators affiliated with each of the hospital-based CPCs. The coordinators are responsible for the coordination and monitoring of the QI activities, including maintaining care coordination between multidisciplinary clinical services for operational integrity with respect to patients with STEMI, conducting performance appraisal and feedback, and training targeted to healthcare professionals and towards health education among community residents. Prior studies have shown that a dedicated coordinator in charge of implementing systematic improvements within hospitals and EMS agencies could play a critical role in maintaining coordination of care [25,26].

Although the NCPCP has made achievements in reducing the reperfusion times, the total delay time (from symptom onset to reperfusion) was about 212.5 min, much longer than the guideline recommends [27]. Onset-to-FMC time accounted for about 53.9% of total delayed time. Patient delay (from onset to when patients enter the EMS system) still appears to hinder the timely delivery of STEMI care. These results may be attributable to low population awareness of signs and symptoms of STEMI or the option of calling EMS, or lack of appropriate EMS response. For a better understanding of coordinated care, we compared the quality of STEMI care by transfer mode. We found that cases that went directly via EMS had a higher increase in most process indicators than cases who self-transported to hospitals. Nevertheless, the proportion of patients presenting directly to PCI-capable hospitals via EMS was much lower than that of self-transported patients, and the proportion declined after the QI initiatives. Therefore, our findings serve to emphasize that we should target specific strategies at the population level, including improving early recognition of STEMI symptoms and awareness of the option of calling EMS among the public, to increase the use of prehospital services and prehospital activation of PCI. To further improve the implementation of the combined measures, EMS should focus more on prehospital processes.

Whereas the specific goals of the NCPCP have been focused on process of care measures, the ultimate goal is to improve clinical outcomes. In the examined time-period, the adjusted in-hospitals mortality increased, despite the improvements in the reperfusion time after the combined measures. This finding can be explained by three factors. First, the major reason for these results was the inclusion of all hospitals, regardless of the duration or the progress of the implementation of QI activities. By providing aggregate results, the absolute potential of the QI activities may not be reflected because some hospitals that participated late in the program were unable to implement improvements within a short timeframe. Second, the program was made available to all secondary and tertiary hospitals, and hospitals continue to join the program in a staggered manner. At the beginning of the program, the majority of registered hospitals were tertiary hospitals with PCI capability. While with the promotion of the program nationwide, an increasing number of secondary hospitals without PCI capability participated in the program, which may lead to worse performance on delivering care and poor improvements in performance. We found that secondary hospitals had longer onset-to-device times than secondary hospitals. Thus, coordinated and hierarchical care between secondary and tertiary hospitals should be enhanced to reduce system delay (from when patients enter the EMS system to reperfusion). Third, the increase of adjusted in-hospital mortality could also be attributable to the enrollment of consecutive patients presenting increasing severity of symptoms, which was documented to be related to increased mortality risk for STEMI. We detected increased rates of cardiac arrest and cardiogenic shock, and an increase in patients with Killip class IV, after the QI initiatives. Before the combined measures, many deaths may have occurred before admission to the hospital of a sudden and unexpected nature, which can be associated with delays in seeking care. With the increasing number of cases entering hospitals since the program, cases with lengthy delays and severe symptoms were recorded on the China CPC Reporting Platform.

We further found regional variations in the quality of STEMI care. Generally, the process of care was better for well-developed urban areas than less-developed suburban areas. Most of the top tertiary hospitals in Suzhou are located in the center of the urban area, and STEMI patients in the suburbs have difficulty transferring to these hospitals in a timely manner. However, the percentage of patients arriving at the first hospital by ambulance after the QI initiative decreased significantly, making it more difficult for patients to reach urban hospitals in time for treatment. Therefore, attention should be paid to increasing the distribution of ambulances in suburban areas, and to improving the prehospital first-aid system in suburban areas. Short-term goals should focus on promoting health education on STEMI rescue and improving public knowledge on the use of EMS at the community level, such us organizing community health missions and training or putting up STEMI rescue related posters. Long-term goals should focus on improving prehospital medical resources to meet the needs of the local population for more effectively mobilizing prehospital resources in the suburban areas. The outcomes of this study could be translated into a systematic solution for improving the quality of STEMI care, generating knowledge about process outcomes and core components that is transferrable, and where local adaptation is needed for replication in other settings. This actionable knowledge is also critical for implementors of scale-up activities in low- and middle-income settings.

The variability in improvement was related primarily to the speed with which districts could implement effective regional systems of STEMI care. A large proportion of work during the implementation of the program was to persuade the majority of health bureaus of districts to put forward a systemic design for establishing a system of coordinated care. The biggest resistance lies in the fragmented financing and supervision for prehospital and hospital care. This highlights the challenge of pursuing such a large-scale implementation during a relatively short time period. Nevertheless, the results from the most improved districts indicated that it was possible to improve reperfusion time step by step according to our approach. From our experience, districts most able to improve reperfusion time had common characteristics, including EMS leadership concentrated to a few dominant agencies, and active engagement by coordinators. Future studies should focus on regional disparities in the performance of the program, to assess the factors among myriad local socioeconomic and political factors causing disparities in quality of care. In addition, more detailed analyses and case studies with low quality of care are needed to identify the interventions that may lead to better outcomes, and that could be applied to the other local settings.

This study had several limitations. First, the study was limited to Suzhou, which limits the generalizability of the results because Suzhou differs from other cities in terms of implementation of the QI initiative, socioeconomic development, population health, and the EMS system. Extrapolation of these results requires further study. Data from other cities should be further explored and verified to improve the accuracy and adaptability of these conclusions. Second, this study was a retrospective study; therefore, we could not establish a causal relationship. Even with PSM, unobserved variables may have biased the results. Follow-up studies with prospective randomized studies are needed. Third, while the implementation of complex strategies that require different steps will become more efficient after a long time of implementation efforts, the lack of mixed analysis of long-term intervention contexts together with recently included hospitals is still a limitation. Follow-up long term analysis is needed in the future.

## 5. Conclusions

This study showed that the combined measures, including the multifaceted QI initiatives and the establishment of an information sharing system, can improve quality of care and showed potential for improving clinical outcomes among STEMI patients. For further promoting the measures, patient delay should be addressed, to reduce the delay for entry into the EMS system, especially in suburban areas, and on transferred-in inpatients. The regional disparities in performance improvement can be related to the speed with which districts could implement effective regional systems of STEMI care. The establishment of coordinated care needs to be accompanied with solving the fragmented situation of the prehospital and hospital care which should be designed specifically to fit into the health systems on a regional basis. The consecutive recruitment of accredited hospitals warrants more efforts to enhance the implementation of the QI initiatives.

## Figures and Tables

**Table 1:** Characteristics of patients with discharged diagnosed STEMI Before and After PSM.

Characteristics of Patients	Before PSM			After PSM		
	Pre-Combined Measures	Post-Combined Measures	*p* Value	Pre-Combined Measures	Post-Combined Measures	*p* Value
Number of hospital admissions	1099	3163		1078	1078	
Age (years) *	61.91 (14.97)	60.40 (14.65)	0.004	61.72 (14.97)	61.82 (14.29)	0.863
Female, n (%)	205 (18.7)	574 (18.1)	0.742	195 (18.1)	190 (17.6)	0.822
Clinical characteristics						
Sustainable chest pain, n (%)	849 (77.3)	2283 (72.2)	0.001	845 (78.4)	848 (78.7)	0.916
Intermittent chest pain, n (%)	160 (14.6)	702 (22.2)	<0.001	155 (14.4)	160 (14.8)	0.807
Chest pain relief, n (%)	27 (2.5)	140 (4.4)	0.005	22 (2.0)	19 (1.8)	0.752
CPR, n (%)	11 (1.0)	40 (1.3)	0.595	11 (1.0)	8 (0.7)	0.645
Heart failure, n (%)	8 (0.7)	41 (1.3)	0.174	8 (0.7)	7 (0.6)	1.000
Cardiogenic shock, n (%)	25 (2.3)	47 (1.5)	0.107	18 (1.7)	11 (1.0)	0.262
Respiratory rate (breaths/min) *	18.11 (3.07)	18.29 (5.68)	0.324	18.11 (3.06)	18.01 (3.65)	0.494
Heart rate (beats/min) *	77.07 (19.61)	78.56 (18.23)	0.021	77.39 (19.49)	78.02 (18.26)	0.437
Systolic blood pressure (mm Hg) *	130.89 (29.68)	130.14 (25.64)	0.424	131.23 (29.39)	131.23 (25.84)	0.998
Diastolic blood pressure (mm Hg) *	81.10 (19.45)	81.08 (17.16)	0.974	81.44 (19.15)	81.91 (17.14)	0.550
Killip class, n (%)			0.257			0.953
I	951 (86.5)	2679 (84.7)		938 (87.0)	940 (87.2)	
II	70 (6.4)	234 (7.4)		68 (6.3)	63 (5.8)	
III	14 (1.3)	64 (2.0)		14 (1.3)	16 (1.5)	
IV	64 (5.8)	186 (5.9)		58 (5.4)	59 (5.5)	

* Mean(SD).

**Table 2:** Changes in reperfusion times and in-hospital mortality among STEMI patients pre- and post-combined measures quarter by hospitals.

Key Performance Indicators	Total Hospitals (*n* = 2056)			Tertiary Hospitals (*n* = 1871)			Secondary Hospitals (*n* = 285)		
	Pre-Combined Measures	Post-Combined Measures	*p* Value	Pre-Combined Measures	Post-Combined Measures	*p* Value	Pre-Combined Measures	Post-Combined Measures	*p* Value
*N*	1078	1078		807	1064		271	14	
Pre-hospital process indicators									
Percent of cases arriving at the first hospital by ambulance, n (%)	100 (9.3)	111 (10.3)	0.464	70 (8.7)	110 (10.4)	0.250	30 (11.2)	1 (7.1)	0.976
Pre-hospital ECGs, n (%)	189 (17.5)	289 (26.8)	<0.001	173 (21.4)	288 (27.1)	0.006	16 (5.9)	1 (7.1)	1.000
Onset-to-FMC (EMS arrival or walk-in to ED) time ≤ 60 min, n (%)	291 (27.6)	328 (32.3)	0.021	220 (27.8)	323 (32.3)	0.047	71 (27.0)	5 (38.5)	0.558
Call-to-EMS time for ambulance transported cases ≤ 15 min, n (%)	47 (97.9)	50 (98.0)	1.000	33 (97.1)	49 (98.0)	1.000	14 (100.0)	1 (100.0)	-
EMS-to-first ECG time for ambulance transported cases ≤ 10 min, n (%)	12 (75.0)	54 (91.5)	0.171	9 (75.0)	53 (91.4)	0.261	3 (75.0)	1 (100.0)	1.000
Ambulance ECG-to-door time for ambulance transported cases ≤ 15 min, n (%)	6 (3.3)	34 (12.1)	0.002	4 (2.4)	34 (12.2)	0.001	2 (13.3)	0 (0.0)	1.000
In-hospital process indicators									
Intensive statin within 24 h, n (%)	610 (73.6)	609 (68.5)	0.024	470 (77.4)	606 (69.2)	0.001	140 (63.1)	3 (23.1)	0.010
Door-to-balloon time ≤ 60 min, n (%)	110 (19.1)	193 (24.0)	0.036	100 (18.9)	193 (24.2)	0.029	10 (21.3)	0 (0.0)	0.582
FMC-to-device time ≤ 90 min, n (%)	273 (44.8)	434 (55.7)	<0.001	251 (44.8)	432 (55.8)	<0.001	22 (44.0)	2 (40.0)	1.000
Onset-to-device time ≤ 120 min, n (%)	80 (13.0)	135 (16.7)	0.064	72 (12.7)	134 (16.6)	0.053	8 (16.3)	1 (20.0)	1.000
Door-to-balloon time, median (q_1_, q_3_)	85.0 [67.0, 108.0]	78.0 [61.5, 92.0]	<0.001	85.0 [66.8, 108.3]	78.0 [61.3, 92.0]	<0.001	87.0 [71.5, 107.0]	79.0 [64.0, 112.0]	0.938
FMC-to-device time, median (q_1_, q_3_)	98.0 [78.0, 132.8]	88.0 [71.0, 124.5]	<0.001	98.0 [78.0, 132.3]	88.0 [71.0, 124.8]	<0.001	96.0 [76.0, 131.3]	101.0 [63.0, 111.0]	0.660
Onset-to-device time, median (q_1_, q_3_)	212.5 [150.8, 325.5]	213.0 [142.0, 372.0]	0.818	215.0 [152.0, 334.0]	212.0 [142.0, 370.0]	0.854	192.0 [136.0, 250.0]	379.0 [351.0, 407.0]	0.098
PCI rate, n (%)	619 (57.4)	823 (76.3)	<0.001	571 (70.8)	818 (76.9)	0.003	48 (17.7)	5 (35.7)	0.182
Outcome indicators									
In-hospital mortality, n (%)	30 (2.9)	29 (2.9)	1.000	24 (3.1)	29 (2.9)	0.897	6 (2.3)	0 (0.0)	1.000
Incidence rate of heart failure, n (%)	132 (15.1)	188 (21.7)	<0.001	91 (13.4)	187 (21.9)	<0.001	41 (20.6)	1 (8.3)	0.508

**Table 3:** Changes in reperfusion times and in-hospital mortality among STEMI patients pre- and post-combined measures quarter by transfer mode.

Key Performance Indicators	Directly by Self			Directly via EMS			Transfer-in			In-Hospital		
	Pre-Combined Measures	Post-Combined Measures	*p* Value	Pre-Combined Measures	Post-Combined Measures	*p* Value	Pre-Combined Measures	Post-Combined Measures	*p* Value	Pre-Combined Measures	Post-Combined Measures	*p* Value
*N*	712	628		100	111		220	323		44	13	
Pre-hospital process indicators												
Onset-to-FMC (EMS arrival or walk-in to ED) time ≤ 60 min, n (%)	158 (22.6)	184 (29.7)	0.004	38 (39.6)	66 (62.9)	0.002	67 (31.0)	67 (24.5)	0.136	28 (68.3)	11 (84.6)	0.430
In-hospital process indicators												
Intensive statin within 24 h, n (%)	396 (71.2)	345 (67.5)	0.212	48 (65.8)	45 (46.9)	0.022	131 (84.0)	208 (77.6)	0.146	33 (78.6)	8 (72.7)	0.994
Door-to-balloon time ≤ 60 min, n (%)	32 (8.8)	84 (17.2)	0.001	10 (17.5)	20 (26.0)	0.343	68 (44.2)	89 (37.4)	0.219	-	-	-
FMC-to-device time ≤ 90 min, n (%)	204 (55.0)	340 (69.8)	<0.001	22 (40.7)	45 (58.4)	0.069	30 (19.0)	46 (22.2)	0.533	17 (63.0)	3 (37.5)	0.383
Onset-to-device time ≤ 120 min, n (%)	46 (12.3)	95 (19.8)	0.005	10 (17.5)	26 (33.3)	0.064	9 (5.7)	12 (4.9)	0.914	15 (57.7)	2 (28.6)	0.346
Door-to-balloon time, median (q_1_, q_3_)	89.0 [75.0, 120.0]	82.0 [68.0, 99.3]	<0.001	87.0 [72.0, 109.0]	71.0 [60.0, 88.0]	0.005	65.0 [47.8, 84.5]	71.5 [49.0, 87.0]	0.158	-	-	-
FMC-to-device time, median (q_1_, q_3_)	88.0 [73.0, 119.0]	80.0 [66.0, 98.0]	<0.001	102.5 [82.3, 127.3]	85.0 [68.0, 105.0]	0.004	121.50 [98.3, 161.8]	136.0 [99.0, 185.0]	0.055	85.0 [71.5, 118.5]	130.0 [74.8, 140.0]	0.467
Onset-to-device time, median (q_1_, q_3_)	215.0 [153.5, 339.5]	203.5 [135.0, 339.0]	0.104	175.0 [140.0, 254.0]	141.5 [115.3, 175.8]	0.010	240.0 [171.0, 347.0]	277.0 [193.0, 465.0]	0.004	96.0 [85.5, 181.8]	140.0 [124.0, 155.0]	0.552
PCI rate, n (%)	375 (52.7)	491 (78.2)	<0.001	57 (57.0)	78 (70.3)	0.063	159 (72.3)	246 (76.2)	0.357	28 (63.6)	8 (61.5)	1.000
Outcome indicators												
In-hospital mortality, n (%)	18 (2.6)	16 (2.8)	1.000	3 (3.2)	2 (1.9)	0.917	7 (3.3)	10 (3.3)	1.000	2 (4.9)	1 (7.7)	1.000
Incidence rate of Heart failure, n (%)	88 (15.0)	102 (20.3)	0.025	18 (24.0)	17 (20.7)	0.765	21 (11.7)	65 (24.3)	0.001	5 (15.2)	3 (27.3)	0.652

**Table 4:** Disparity between urban and suburban area in reperfusion times and in-hospital mortality among STEMI patients.

Key Performance Indicators	Total Hospitals (n = 2056)			Urban Area (n = 1201)			Suburban Area (n = 955)		
	Pre-Combined Measures	Post-Combined Measures	*p* Value	Pre-Combined Measures	Post-Combined Measures	*p* Value	Pre-Combined Measures	Post-Combined Measures	*p* Value
*N*	1078	1078		524	677		554	401	
Pre-hospital process indicators									
Percent of cases arriving at the first hospital by ambulance, n (%)	100 (9.3)	111 (10.3)	0.464	41 (7.8)	89 (13.2)	0.004	59 (10.7)	22 (5.5)	0.006
Pre-hospital ECGs, n (%)	189 (17.5)	289 (26.8)	<0.001	74 (14.1)	129 (19.1)	0.029	115 (20.8)	160 (39.9)	<0.001
Onset-to-FMC (EMS arrival or walk-in to ED) time ≤ 60 min, n (%)	291 (27.6)	328 (32.3)	0.021	138 (27.0)	224 (36.1)	0.001	153 (28.2)	104 (26.5)	0.601
Call-to-EMS time for ambulance transported cases ≤ 15 min, n (%)	47 (97.9)	50 (98.0)	1.000	17 (94.4)	45 (97.8)	1.000	30 (100.0)	5 (100.0)	-
EMS-to-first ECG time for ambulance transported cases ≤ 10 min, n (%)	12 (75.0)	54 (91.5)	0.171	6 (66.7)	51 (91.1)	0.128	6 (85.7)	3 (100.0)	1.000
Ambulance ECG-to-door time for ambulance transported cases ≤ 15 min, n (%)	6 (3.3)	34 (12.1)	0.002	5 (6.9)	33 (27.5)	0.001	1 (0.9)	1 (0.6)	1.000
In-hospital process indicators									
Intensive statin within 24 h, n (%)	610 (73.6)	609 (68.5)	0.024	260 (67.7)	315 (54.3)	<0.001	350 (78.7)	294 (95.1)	<0.001
Door-to-balloon time ≤ 60 min, n (%)	110 (19.1)	193 (24.0)	0.036	57 (19.4)	113 (21.5)	0.535	53 (18.9)	80 (28.9)	0.007
FMC-to-device time ≤ 90 min, n (%)	273 (44.8)	434 (55.7)	<0.001	152 (49.0)	294 (59.5)	0.005	121 (40.3)	140 (49.1)	0.040
Onset-to-device time ≤ 120 min, n (%)	80 (13.0)	135 (16.7)	0.064	49 (15.7)	98 (18.7)	0.304	31 (10.2)	37 (12.9)	0.369
Door-to-balloon time, median (q_1_, q_3_)	85.00 [67.00, 108.00]	78.00 [61.50, 92.00]	<0.001	84.0 [67.0, 106.8]	78.0 [64.0, 90.0]	0.004	85.0 [66.0, 108.0]	79.0 [58.0, 98.0]	0.003
FMC-to-device time, median (q_1_, q_3_)	98.00 [78.00, 132.75]	88.00 [71.00, 124.50]	<0.001	93.5 [75.0, 126.0]	85.0 [68.0, 123.0]	0.003	99.5 [81.0, 140.0]	92.0 [77.0, 130.0]	0.058
Onset-to-device time, median (q_1_, q_3_)	212.50 [150.75, 325.50]	213.00 [142.00, 372.00]	0.818	204.0 [141.0, 346.0]	210.0 [138.8, 368.5]	0.867	220.0 [157.5, 315.5]	221.0 [151.0, 382.3]	0.680
PCI rate, n (%)	619 (57.4)	823 (76.3)	<0.001	314 (59.9)	536 (79.2)	<0.001	305 (55.1)	287 (71.6)	<0.001
Outcome indicators									
In-hospital mortality, n (%)	30 (2.9)	29 (2.9)	1.000	16 (3.3)	19 (3.0)	0.905	14 (2.6)	10 (2.8)	1.000
Incidence rate of Heart failure, n (%)	132 (15.1)	188 (21.7)	<0.001	60 (14.2)	138 (24.5)	<0.001	72 (15.9)	50 (16.6)	0.872

## Data Availability

The data presented in this study are available on request from the corresponding author. The data are not publicly available due to the license of National Chest Pain Center (CPC) Data Reporting Database.

## Supplementary Materials

The following are available online at https://www.mdpi.com/article/10.3390/ijerph18116045/s1, Supplement Table S1: Definition and measures of quality metrics. Supplement Table S2: Definition and measures of confounding variables for propensity score matching. Supplement Figure S1: Mirrored Histogram before (A) and after (B) propensity score matching. X axis is the number of patients in each group. Y axis is the propensity score. The blue bar presents the pre-combined measures group and the red bar for the post-combined measures group.
