# Supplementary material for: Health Care Quality Improvement for ST-Segment Elevation Myocardial Infarction: A Retrospective Study Based on Propensity-Score Matching Analysis
Source: Int J Environ Res Public Health. 2021 Jun 4;18(11):6045. doi: 10.3390/ijerph18116045 (PMC8200089; doi:10.3390/ijerph18116045)
Supplement: Supplementary file 1 [file ijerph-18-06045-s001.zip › ijerph-1232219-supplementary.pdf]

Supplementary Materials:

Supplementary Table 1 Definition and measures of confounding variables for propensity score matching

	Definition	Measures
**Patient demographics**		
Age	Age of patients	Numeric (year)
Sex	Sex of patients	1=Man，2=Woman
**Vital sighs**		
Respiratory rate	Patient's breath rate per minute during physical examination	Numeric (breaths/min)
Heart rate	Patient's heart rate per minute during physical examination	Numeric (beats/min)
Systolic blood pressure	Patient's Systolic blood pressure during physical examination	Numeric（mmHg）
Diastolic blood pressure	Patient's Diastolic blood pressure during physical examination	Numeric（mmHg）
Killip grade	Patient’s Killip grade	1=Level I，2= Level II<br>3=Level III，4= Level IV
**Clinical characteristics**		
Sustainable chest pain	Sustainable chest pain showed on patient	0=No, 1=Yes
Intermittent chest pain	Intermittent chest pain showed on patient	0=No, 1=Yes
Chest pain relief	Patient’s chest pain relief	0=No, 1=Yes
CPR	CPR is performed on the patient	0=No, 1=Yes
Heart failure	Heart failure occurs in patients	0=No, 1=Yes
Cardiogenic shock	Cardiogenic shock occurs in patients	0=No, 1=Yes

Supplementary Table 2 Definition and measures of quality metrics

	Definition	Measures
**Pre-hospital process indicators**		
Onset-to-FMC (EMS arrival or walk-in to ED) time≤60 min, n (%)	Percentage of patient onset time to first medical contact (FMC) time less than or equal 60 minutes	= (Cases Onset-to-FMC ≤60 min)/(Cases with onset time and FMC time)
Call-to-EMS time ambulance transported cases ≤15 min, n (%)	Percentage of ambulance transported patient call time to emergency medical service (EMS) time less than or equal 15 minutes	= (Cases Call-to-EMS ≤15 min)/(Cases with call time and EMS time)
EMS-to-first ECG time for ambulance transported cases≤10 min, n (%)	Percentage of ambulance transported patient emergency medical service (EMS) time to pre-hospital ECG time less than or equal 10 minutes	= (Cases EMS-to-first ECG ≤10 min)/(Cases with EMS time and pre-hospital ECG time)
Ambulance ECG to door time for ambulance transported cases≤15 min, n (%)	Percentage of ambulance transported patient pre-hospital ECG time to door time less than or equal 15 minutes	= (Cases Ambulance ECG to door time ≤15 min)/(Cases with pre-hospital ECG time and door time)
Percent of cases arriving at the first hospital by ambulance, n (%)	Percentage of cases arriving at the first hospital by ambulance among all transfer mode	= (Cases arriving at the first hospital by ambulance)/(All cases)
Pre-hospital ECGs, n (%)	Percentage of cases use pre-hospital ECGs among all ambulance transported cases	= (Cases with pre-hospital ECG)/(Cases arriving at the first hospital by ambulance)
**Hospital process indicators**		
Intensive statin within 24 hours, n (%)	Percentage of patient use statin within 24 hours	= (Cases use statin within 24 hours)/(All cases)
Door-to-balloon time ≤60 min, n (%)	Percentage of PCI patient door time to balloon time less than or equal 60 minutes	= (PCI cases door-to-balloon time ≤ 60 min)/(PCI Cases with door time and balloon time)
FMC-to-device time ≤90 min, n (%)	Percentage of PCI and thrombolysis patient first medical contact time to device time less than or	= (PCI and thrombolysis cases FMC-to-device time ≤90 min)/(PCI and thrombolysis Cases

	equal 90 minutes	with FMC time and device time)
Onset-to-device time ≤120 min, n (%)	Percentage of PCI and thrombolysis patient onset time to device time less than or equal 90 minutes	= (PCI and thrombolysis cases onset-to-device time ≤120 min)/(PCI and thrombolysis Cases with onset time and device time)
Door-to-balloon time, median (q_1, q_3)	Median of door to balloon time for PCI patient	= Balloon time – door time (min)
FMC-to-device time, median (q_1, q_3)	Median of FMC to device time for PCI patient	= device time – FMC time(min)
Onset-to-device time, median (q_1, q_3)	Median of onset to device time for PCI patient	= device time – onset time(min)
PCI rate, n (%)	Percentage of patients take PCI among all patients	= (Cases of PCI)/(All cases)
**Outcome indicators**		
In-hospital mortality, n (%)	Percentage of patient died in hospital	= (Cases of died patient)/(All cases)
Incidence rate of Heart failure, n (%)	Percentage of patient occur heart failure in hospital	= (Cases occur heart failure in hospital)/(All cases)

Supplementary Figure 1 Mirrored Histogram before (A) and after (B) propensity score matching. X axis is the number of patients in each group. Y axis is the propensity score. The blue bar presents the pre-combined measures group and the red bar for the post-combined measures group.

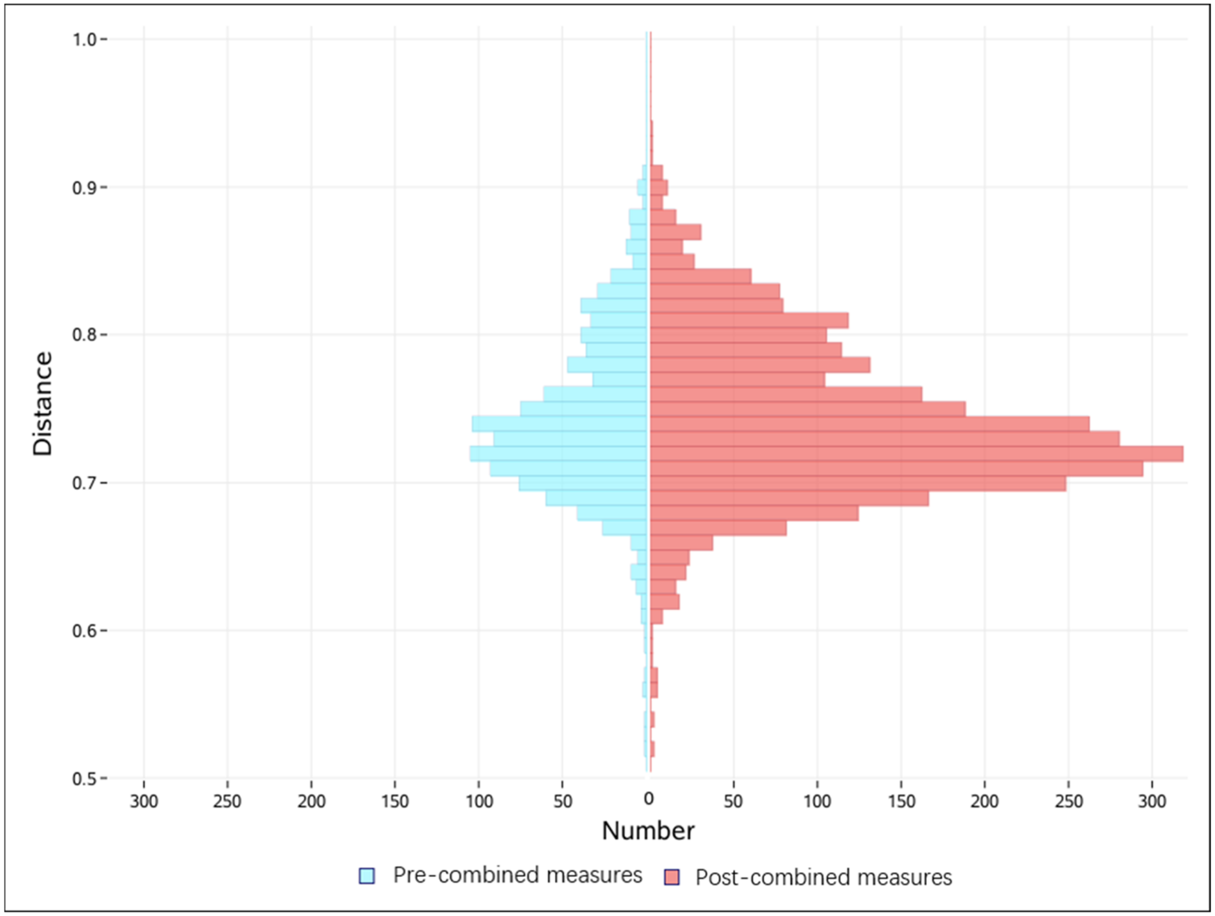

Supplement Figure 1A

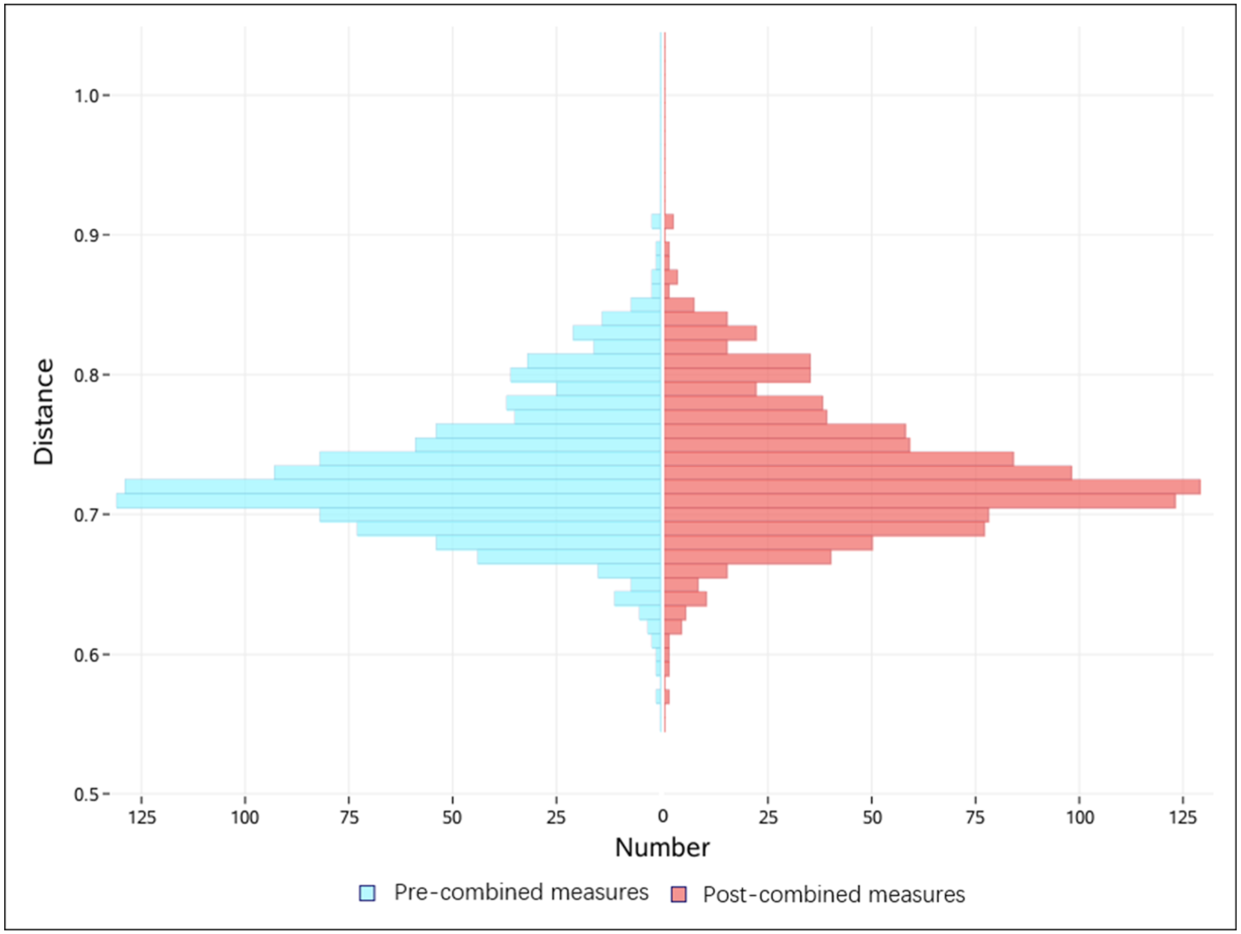

Supplement Figure 1B
